# Changes in Left Ventricular Afterload Following Weaning From Cardiopulmonary Bypass Proved Useful for the Anesthetic Management of Aortic Valve Replacement: A Case Report

**DOI:** 10.7759/cureus.114078

**Published:** 2026-08-06

**Authors:** Keisuke Sumii, Tomohisa Tamai, Takumi Yamamoto

**Affiliations:** 1 Anesthesiology, Saitama Sekishinkai Hospital, Sayama, JPN; 2 Cardiovascular Diseases, Saitama Sekishinkai Hospital, Sayama, JPN

**Keywords:** aortic valve replacement, cardiopulmonary bypass, mechanical valve, physiological regurgitation, pressure gradient

## Abstract

During aortic valve replacement, after weaning from the cardiopulmonary bypass (CPB), systemic vascular resistance, which constitutes the left ventricular afterload, fluctuates significantly due to systemic inflammatory responses, changes in body temperature, and the use of vasoconstrictors. Particularly in hypertrophic hearts that have adapted to increased afterload for a long time, a sudden increase in afterload following weaning from CPB carries a risk of inducing left heart failure or prosthetic valve failure. With mechanical prosthetic valves, a slight physiological regurgitation is observed during valve closure. In this case, by using pulse Doppler echocardiography and transesophageal echocardiography to monitor this physiological regurgitation in real time, we were able to rapidly assess changes in left ventricular afterload and accurately manage the administration of vasoactive agents; we therefore report on the clinical course of this case.

## Introduction

In anesthetic management during the perioperative period of aortic valve replacement, it is important to focus circulatory management on the left ventricle, which is extremely vulnerable to sudden changes in afterload following valve replacement due to myocardial hypertrophy and altered wall stress resulting from long-term stenosis and regurgitation [1,2,3]. It is well known that when weaning from cardiopulmonary bypass (CPB), rapid fluctuations in systemic vascular resistance often occur due to the invasiveness of surgery and rewarming following hypothermia, and that afterload mismatch can easily lead to heart failure; therefore, it is extremely useful to quantitatively assess not only left ventricular systolic function but also changes in the left ventricular afterload [4]. Therefore, appropriate assessment of left ventricular afterload and fine-tuning of vasodilators and inotropic agents based on this assessment are essential [5,6].

Traditionally, continuous cardiac output measurement via a pulmonary artery catheter and observation of left ventricular end-diastolic volume and wall motion via transesophageal echocardiography have been widely used to assess intraoperative left ventricular afterload and stroke volume. However, there is a time lag in the calculation of systemic vascular resistance using a pulmonary artery catheter, and geometric assessments via transesophageal echocardiography often fail to capture accurate, real-time changes in afterload during situations involving drastic changes in cardiac position, such as when weaning from the CPB or during tachycardia [7,8,9].

It is known that, due to their structural design, mechanical prosthetic valves generate a small amount of physiological regurgitation upon closure. The signal from this physiological regurgitation can be captured extremely clearly and in real time as a regurgitation velocity using the pulse Doppler method of transesophageal echocardiography. Since the regurgitation velocity during diastole reflects the pressure difference between aortic pressure and left ventricular diastolic pressure, detailed and continuous monitoring of these dynamic changes can serve as a practical real-time indicator of left ventricular afterload [10].

Reports of actively applying physiological regurgitation of a mechanical prosthetic valve as an indicator of left ventricular afterload to anesthesia and circulatory management during an extremely unstable period, such as immediately after weaning from CPB, as in this case, are extremely rare. In this report, we present specific hemodynamic data and echocardiographic findings and report on the usefulness of intraoperative anesthesia management using physiological regurgitation of a prosthetic valve as an indicator, incorporating a review of the literature.

## Case presentation

A male patient in his 50s (height: 164 cm; body weight: 94 kg) with a history of non-ST-elevation myocardial infarction (drug-eluting stent placed in the right coronary artery three years ago) and hypertension presented with shortness of breath on exertion as his chief complaint. Preoperative transthoracic echocardiography revealed severe mitral and aortic valve stenosis (aortic valve orifice area: 0.71 cm², aortic valve orifice area index: 0.35 cm²/m², maximum aortic blood flow velocity: 4.0 m/sec, mean aortic pressure gradient: 40 mmHg) and severe functional mitral regurgitation. Coronary angiography revealed a 90% stenosis in the left coronary artery (LCA 9). Aortic valve replacement, mitral valve repair, and coronary artery bypass grafting were scheduled to be performed via a median sternotomy.

**Figure 1:**
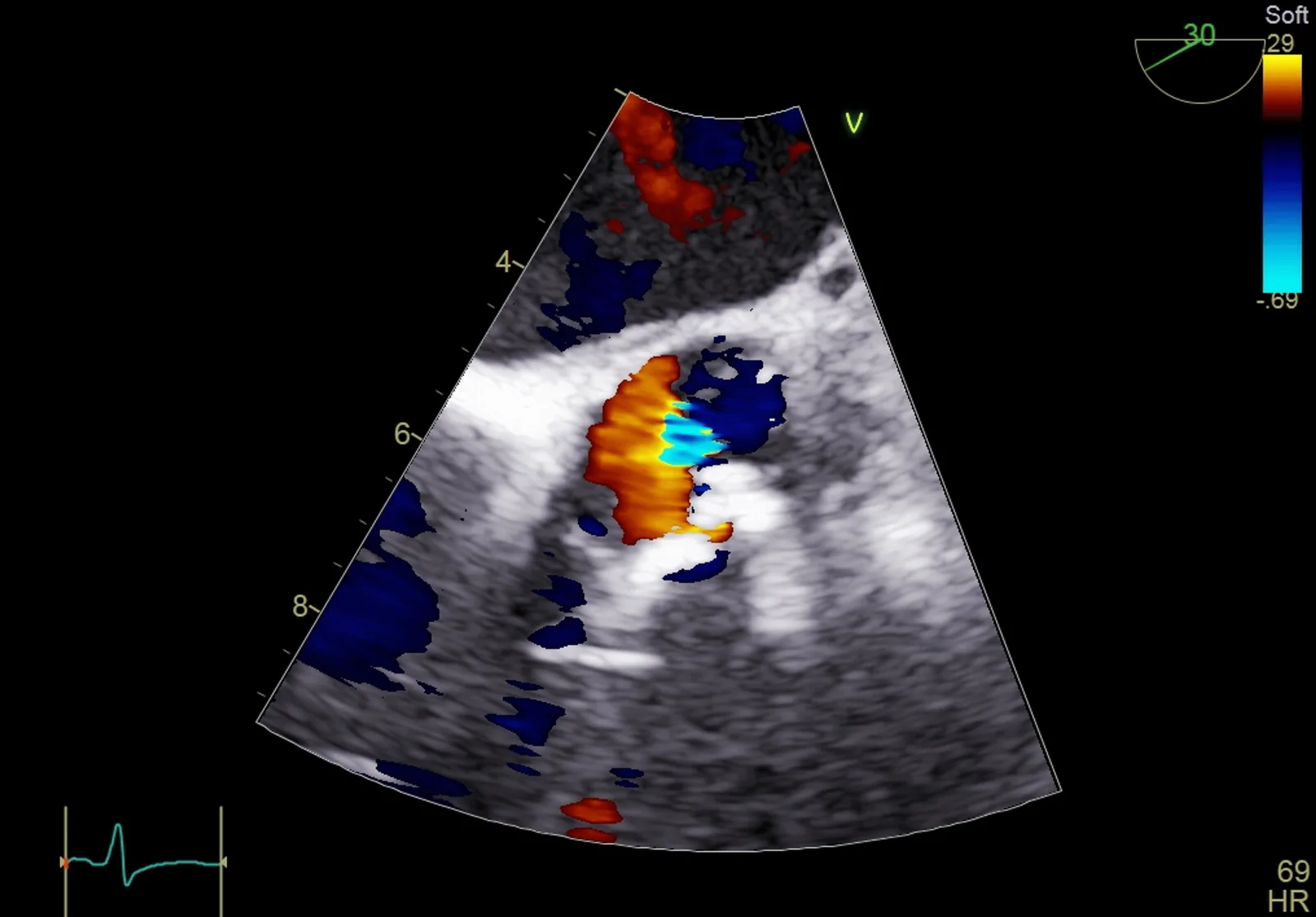
Transesophageal echocardiogram showing a short-axis view of the aortic valve (systolic, color mode). The right and left coronary cusps have fused to form a raphe (arrow). The echogenicity in this area is increased, and the areas where the color echo is not visualized along the aortic annulus correspond to the regions where the fused cusps are not neatly accommodated.

Induction of anesthesia was performed with midazolam, fentanyl, and rocuronium, and a transesophageal echocardiography probe was promptly inserted. A bicuspid aortic valve, in which the right and left coronary cusps formed a raphe, was observed on the short-axis view of the aortic valve (Figure 1).

After establishing the CPB, the aorta was occluded, the calcified aortic valve was resected, and a 23-mm mechanical heart valve (ON-X®) was implanted. For the mitral valve, the annulus was reinforced using a ring. For the coronary arteries, a bypass graft was created using the saphenous vein from the aortic root to the left coronary artery diagonal branch. After weaning from the CPB, the mobility of both the aortic valve and the implanted prosthetic valve was good, and no obvious paravalvular regurgitation was observed. Mitral regurgitation had resolved, left ventricular wall motion was normal, and hemodynamic status was stable. To estimate left ventricular end-diastolic pressure, the E wave was measured from trans-mitral blood flow using pulsed-wave Doppler, and the e’ wave was measured from the mitral valve displacement velocity on the left ventricular lateral wall using tissue Doppler.

In preparation for weaning from the CPB, we initiated administration of dobutamine 1.1 γ, norepinephrine 0.05 γ, and nitroglycerin 0.18 γ. After the aortic cross-clamp was released, atrial fibrillation was observed once; electrical defibrillation at 20 J was performed, resulting in restoration of sinus rhythm, and no further arrhythmias were observed thereafter.

**Figure 2:**
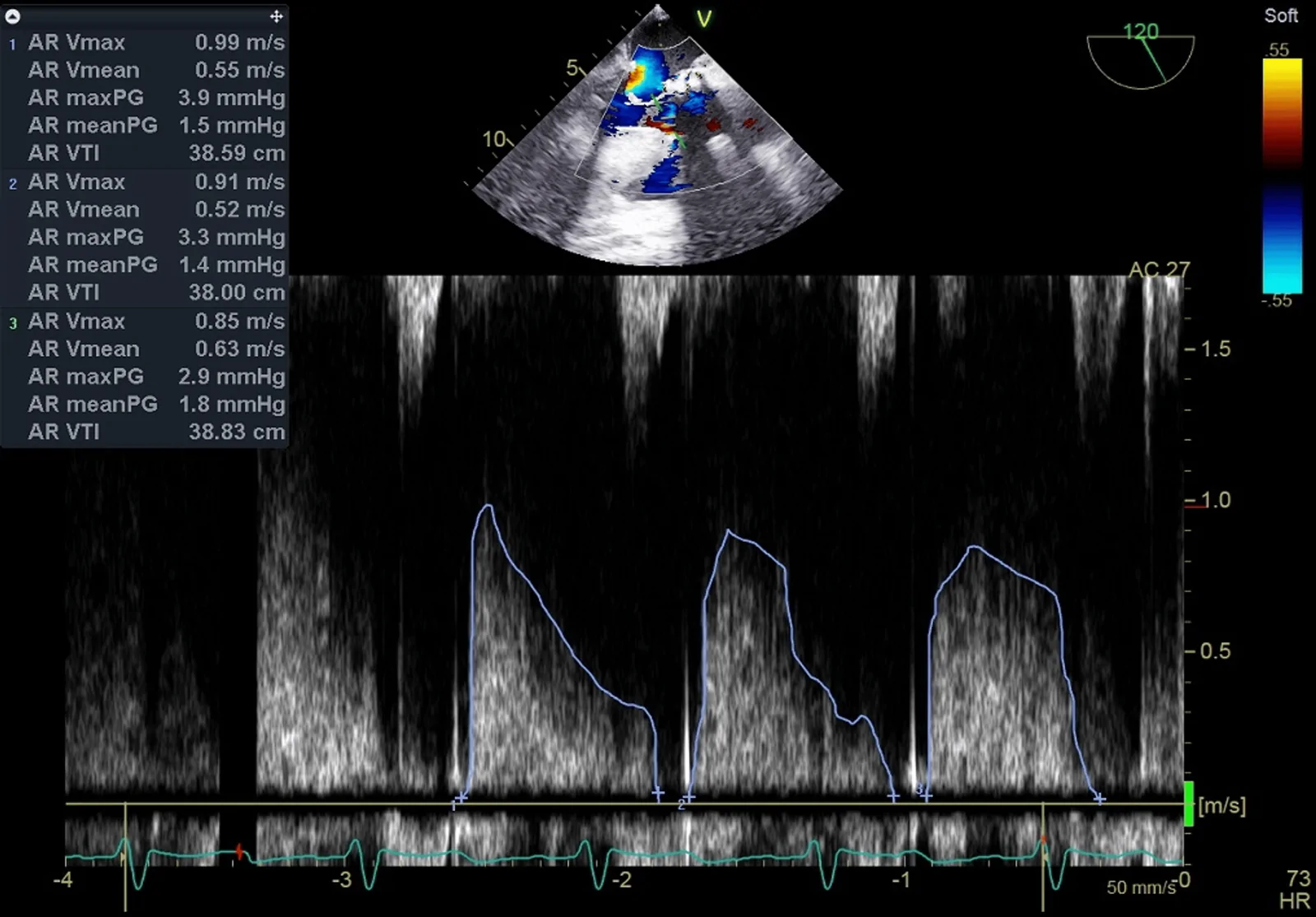
Transesophageal echocardiogram showing a long-axis view of the aortic valve immediately after weaning from the CPB. We used color Doppler imaging to trace the physiological regurgitation flowing from a mechanical prosthetic valve implanted at the aortic valve site into the left ventricle during diastole. By tracing the three physiological regurgitation waves, we measured the average pressure difference, which was found to be 1.5, 1.4, and 1.8 mmHg; we calculated the average value to be 1.57 mmHg.

Immediately after weaning from the CPB, following sternal closure, and at the end of surgery, we used color Doppler transesophageal echocardiography to trace consecutive three heartbeats of physiological regurgitation from the mechanical prosthetic valve and calculated the mean aortic-left ventricular pressure gradient with the aortic valve long-axis view (120 degrees). The invasive arterial pressure was 83/44 mmHg, the heart rate was 70 beats/min, the central venous pressure was 10 mmHg, and the mean pressure gradient between the aorta and the left ventricle was 1.57 mmHg (Figure 2).

**Figure 3:**
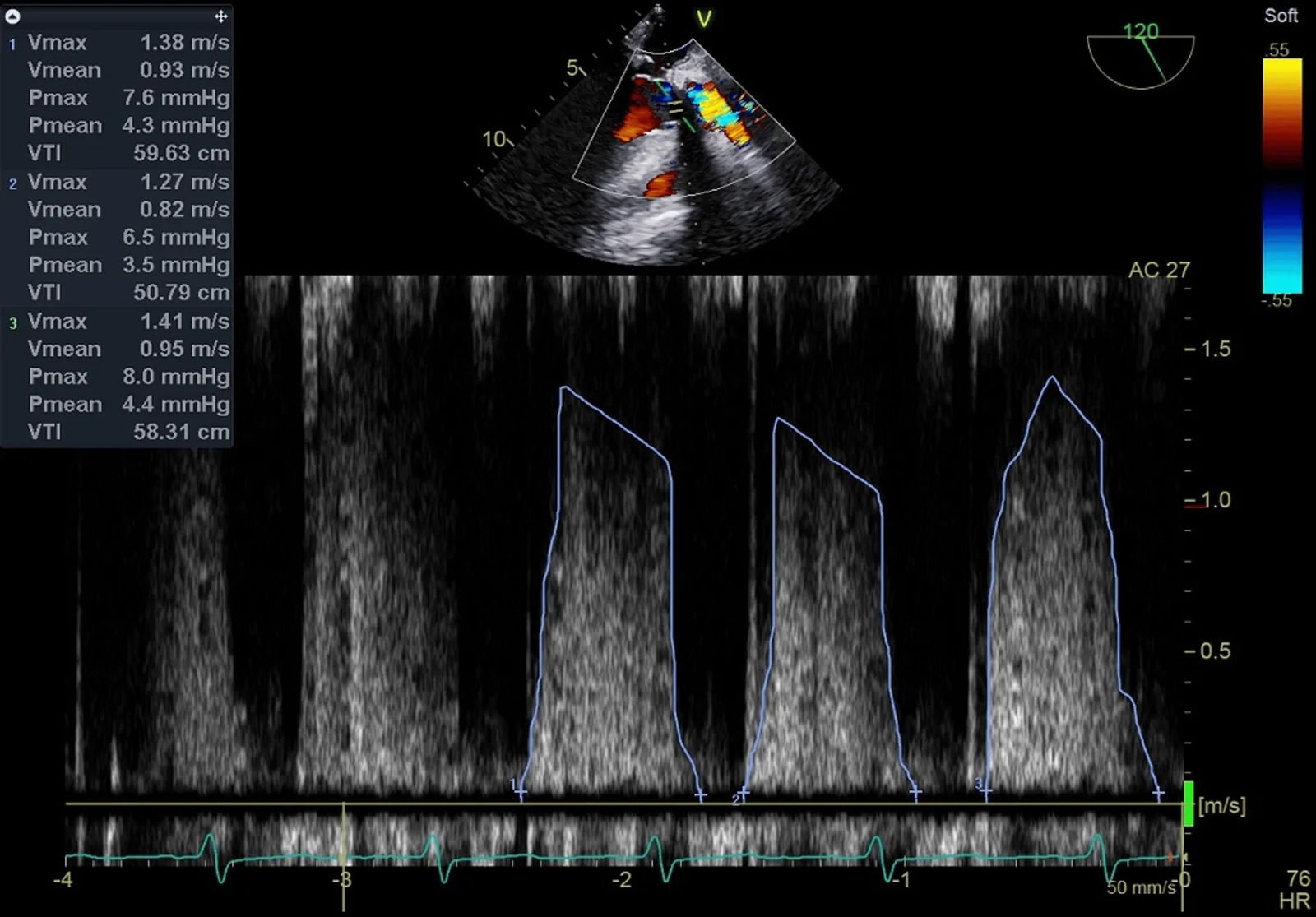
Transesophageal echocardiogram showing a long-axis view of the aortic valve after sternal closure. We used color Doppler imaging to trace the physiological regurgitation flowing from a mechanical prosthetic valve implanted at the aortic valve site into the left ventricle during diastole. By tracing the three physiological regurgitation waves, we measured the average pressure difference, which was found to be 4.3, 3.5, and 4.4 mmHg; we calculated the average value to be 4.07 mmHg.

At that time, the average velocity of the E wave (transmitral blood flow) over three heartbeats was 1.27 m/sec, and the average velocity of the e’ wave (mitral valve displacement velocity at the left ventricular lateral wall) over three heartbeats was 0.02 m/sec. After sternal closure, the invasive arterial pressure was 106/54 mmHg, the heart rate was 74 beats/min, the central venous pressure was 11 mmHg, and the mean aortic-left ventricular pressure gradient was 4.07 mmHg (Figure 3).

**Figure 4:**
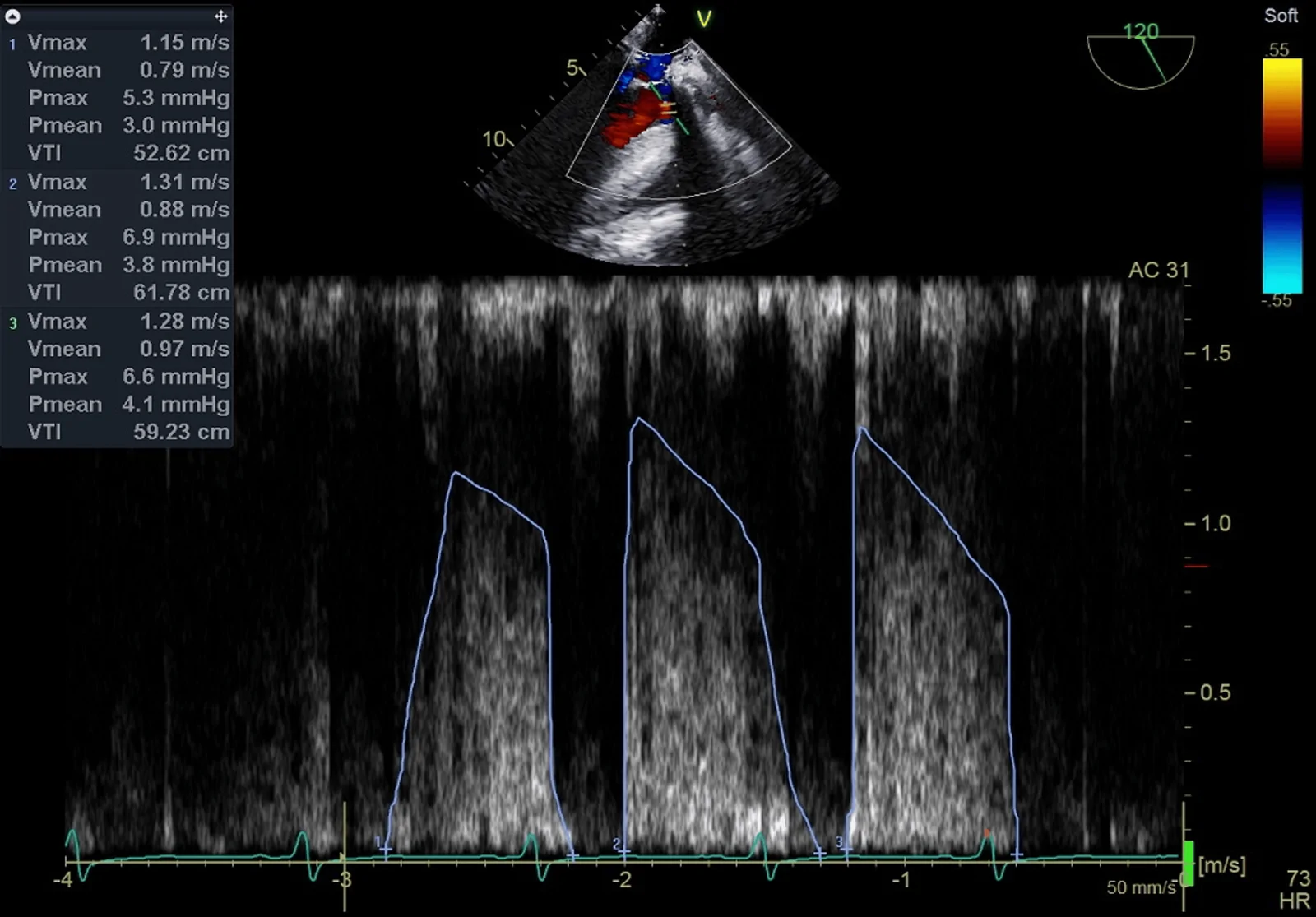
Transesophageal echocardiogram showing a long-axis view of the aortic valve at the end of surgery. We used color Doppler imaging to trace the physiological regurgitation flowing from a mechanical prosthetic valve implanted at the aortic valve site into the left ventricle during diastole. By tracing the three physiological regurgitation waves, we measured the average pressure difference, which was found to be 3.0 mmHg, 3.8 mmHg, and 4.1 mmHg; we calculated the average value to be 3.63 mmHg.

At that time, the average velocity of the E wave (transmitral blood flow) over three heartbeats was 1.24 m/sec, and the average velocity of the e’ wave (mitral valve displacement velocity at the left ventricular lateral wall) over three heartbeats was 0.02 m/sec. Because an increase in the mean pressure gradient between the aorta and the left ventricle was observed, nicardipine (0.2 mg) was administered intravenously as needed, and the nitroglycerin dose was increased to 0.27 γ; as a result, the gradient decreased to 3.63 mmHg at the end of the surgery (Figure 4).

The invasive arterial blood pressure was 110/55 mmHg, the heart rate was 75 beats/min, and the central venous pressure was 11 mmHg. At that time, the average velocity of the E wave in trans-mitral blood flow over three heartbeats was 1.09 m/sec, and the average velocity of the e’ wave, representing mitral valve displacement at the left ventricular lateral wall, over three heartbeats was 0.02 m/sec.

**Figure 5:**
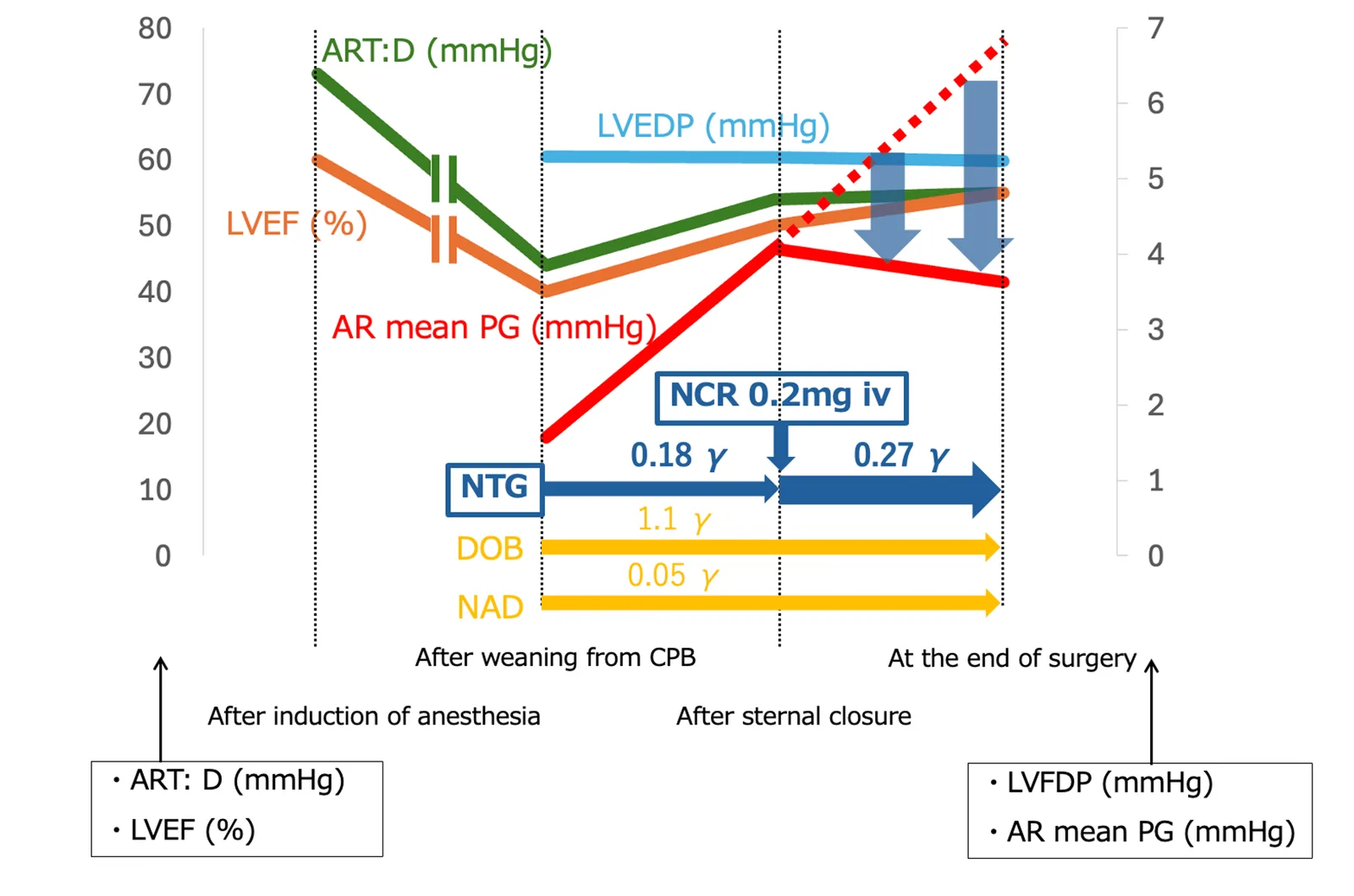
A line graph shows the diastolic blood pressure measured by intraoperative invasive arterial pressure monitoring (ART:D, green), the left ventricular ejection fraction (LVEF, orange) measured by TEE, the left ventricular end-diastolic pressure (LVEDP, light blue), and the mean pressure gradient of the physiological regurgitation of the mechanical prosthetic valve (AR mean PG, red) measured by TEE. Measurements were taken on four occasions: after induction of anesthesia, after weaning from the CPB, after sternal closure, and at the end of surgery. The units for ART:D and LVEF are mmHg and %, respectively, and they are aligned on the left axis. The units for LVEDP and AR mean PG are both mmHg, and they are aligned on the right axis. While the mean pressure gradient between the aorta and the left ventricle was 1.57 mmHg after weaning from the CPB, it increased to 4.07 mmHg after sternal closure. Therefore, 0.2 mg of nicardipine was administered intravenously after sternal closure, and the nitroglycerin dose was increased from 0.18 γ to 0.27 γ; as a result, the pressure gradient had decreased to 3.63 mmHg by the end of surgery. The left ventricular end-diastolic pressure did not change significantly from the time of weaning from the CPB until the end of surgery. Diastolic blood pressure and LVEF increased slightly from the time of weaning from the CPB until the end of surgery. LVEF: left ventricular ejection fraction, TEE: transesophageal echocardiogram, LVEDP: left ventricular end-diastolic pressure, CPB: cardiopulmonary bypass

To estimate left ventricular pressure immediately after weaning from the CPB, after sternal closure, and at the end of surgery, using previously reported formulas, we measured the average velocity of the mitral regurgitation E-wave over three heartbeats using pulse Doppler and the mitral valve displacement velocity e’ wave of the left ventricular lateral wall over three heartbeats using tissue Doppler. The left ventricular end-diastolic pressure was 5.29, 5.28, and 5.24 mmHg, respectively, showing no significant variation (Figure 5).

No symptoms of heart failure or arrhythmias were observed postoperatively, and the prosthetic valve function remained stable. On the first postoperative day, vasodilators and all catecholamines were discontinued, and the patient was extubated. On the 14th postoperative day, the patient was discharged in good health without complications.

## Discussion

In this case, evaluating the physiological regurgitation of a mechanical heart valve using transesophageal echocardiography with pulse Doppler allowed for real-time assessment of left ventricular afterload, which proved useful for anesthetic management.

Following aortic valve replacement, the heart is relieved of the high left ventricular afterload caused by aortic stenosis to which it had been exposed for a long period; however, left ventricular compliance temporarily decreases further due to myocardial ischemia and edema caused by cardiac arrest and the CPB, as well as abrupt hemodynamic changes [11]. Consequently, the heart is highly vulnerable to a sudden increase in left ventricular afterload following weaning from the CPB; such a sudden increase can lead to a decrease in stroke volume, an elevation in left ventricular end-diastolic pressure, and even acute heart failure [2]. Therefore, it is crucial to rapidly assess the rise in afterload and initiate therapeutic interventions. Since the average value of data from a few minutes ago is displayed, afterload is typically assessed using a Swan-Ganz catheter to measure cardiac output and calculate systemic vascular resistance based on mean arterial pressure and central venous pressure; however, this is an intermittent assessment, and there is a time lag before the calculated output is available [7]. Furthermore, while continuous cardiac output monitoring via waveform analysis of invasive arterial pressure is also useful, it is considered prone to errors immediately after weaning from the CPB and susceptible to the effects of damping, which is a distortion of the arterial pressure waveform, when peripheral vascular compliance is extremely low [12]. As demonstrated in this case, even if the mean pressure gradient between the aorta and the left ventricle itself has not yet reached the therapeutic range, left ventricular afterload can undergo rapid changes following weaning from the CPB; therefore, a prompt response is required even to slight increases or decreases.

Physiological regurgitation observed in mechanical prosthetic valves serves not only to maintain clearance in the valve’s moving parts but also to prevent “stuck valve” - a condition in which the valve becomes immobilized due to thrombus formation caused by blood stasis at the pivot point [13,14]. For this reason, mechanical prosthetic valves possess a “washing effect.” Physiological regurgitation waves originate from the inner surface of the valve and are characterized by narrow, sharp, bilaterally symmetrical signals. The maximum blood flow velocity of this regurgitant flow follows the simplified Bernoulli’s equation (ΔP = 4V²) and depends on the difference between aortic diastolic pressure and left ventricular diastolic pressure [15]. As aortic pressure rises due to increased left ventricular afterload, the Vmax of physiological regurgitation increases, and the brightness of the pulse Doppler waveform characteristically increases. In this case, by observing the physiological regurgitation wave from a mechanical prosthetic valve using pulse Doppler, we were able to detect in real time a change in the mean aortic-to-left ventricular pressure gradient from 1.57 mmHg to 4.07 mmHg. This allowed us to reduce left ventricular afterload with vasodilators, such as nicardipine and nitroglycerin, before cardiac function deteriorated, thereby maintaining appropriate hemodynamic stability. Therapeutic intervention was performed even though the increase in pressure gradient was minimal. This was because we believed that left ventricular afterload could change rapidly immediately after weaning from CPB, and we therefore determined that preventive therapeutic intervention was necessary.

However, it is important to remember that the 2024 American Society of Echocardiography guidelines for the functional evaluation of artificial heart valves state that eccentric regurgitant jets from mechanical heart valves do not have a fixed pattern and are often difficult to quantify, so caution is required when evaluating them [16]. It is also necessary to be cautious because various confounding factors must be taken into account. These include left ventricular preload, heart rate, and the characteristics of the prosthetic valve, all of which are essential for an accurate assessment of left ventricular afterload. As the left ventricular preload increases, the left ventricular internal diameter also increases; therefore, even if aortic pressure remains constant, left ventricular afterload increases in tandem with the rise in systolic wall stress [17,18]. Furthermore, as the heart rate increases, the diastolic phase becomes shorter, reducing the time for blood to flow from the aorta to the peripheral arteries. This leads to an increase in diastolic aortic pressure, which in turn increases left ventricular afterload [19]. In addition, even when a prosthetic valve is functioning normally, its effective orifice area is smaller than that of a native valve, resulting in a constant pressure gradient [20]. It is also important to note that if the angle between the ultrasound beam and the blood flow is misaligned, the measured flow velocity may be underestimated compared to the actual flow velocity. Furthermore, since the findings of this study are limited to a case report, their reproducibility must be demonstrated in future large studies.

## Conclusions

In the management of anesthesia following aortic valve replacement, detailed observation of the physiological regurgitation waveforms of the prosthetic valve using intraoperative transesophageal echocardiography may serve as a valuable indicator for real-time, noninvasive detection of abrupt changes in left ventricular afterload following weaning from the CPB.

This approach was considered to contribute significantly to prompt anesthesia and pharmacological management aimed at optimally maintaining postoperative hemodynamic stability.
